# Vitamin-D status and neurodevelopment and growth in young north Indian children: a secondary data analysis

**DOI:** 10.1186/s12937-017-0285-y

**Published:** 2017-09-18

**Authors:** Ranadip Chowdhury, Sunita Taneja, Nita Bhandari, Ingrid Kvestad, Tor A. Strand, Maharaj Kishan Bhan

**Affiliations:** 1 Centre for Health Research and Development, Society for Applied Studies, New Delhi, India; 2 Department of Global Public Health and Primary Care, University of Bergen, Bergen, Norway; 3 Regional Centre for Child and Youth Mental Health and Child Welfare, West, Uni Research Health, Bergen, Norway; 4 Department of Research, Innlandet Hospital Trust, Brumunddal, Norway; 5 Centre for Intervention Science in Maternal and Child Health, Centre for International Health, University of Bergen, Bergen, Norway; 6 Indian Institute Technology - Delhi, New Delhi, 110016 India; 7 Knowledge Integration and Translational Platform (KnIT), Biotechnology Industry Research Assistance Council (BIRAC), New Delhi, India

**Keywords:** Vitamin-D, ASQ-3, Neurodevelopment, Physical growth, Young north Indian children

## Abstract

**Background:**

Vitamin-D deficiency has been linked with impaired development in animal studies; however, the evidence from human studies is scanty. Evidence as to whether vitamin-D deficiency during early childhood affects growth is also limited and conflicting. We examined the extent to which vitamin-D deficiency (<10 ng/ml) is associated with neurodevelopment and physical growth in young children.

**Methods:**

We used data from a randomized controlled trial (RCT) of daily folic acid and/ or vitamin B12 supplementation for six months in children aged 6 to 30 months conducted in Delhi, India. We measured vitamin-D status and  neurodevelopment by the Ages and Stages Questionnaire-3 (ASQ-3) at 12 to 36 months of age. Multiple logistic and linear regressions were used to examine the association between vitamin-D deficiency at baseline and neurodevelopment and growth 6 months follow-up.

**Results:**

25-hydroxy-vitamin-D (25OHD) concentration was measured at baseline for 960 (96%) children. Of these, 331 (34.5%) children were vitamin-D deficient. The total and subscale (except for the Personal social scale) ASQ-3 scores, were not different between the vitamin-D deficient and non-deficient children. Vitamin-D deficiency was also not associated with physical growth at baseline and at follow -up.

**Conclusion:**

Our data do not support the hypothesis that vitamin-D deficiency is associated with poor growth and neurodevelopment.

**Trial registration:**

NCT00717730 and CTRI/2010/091/001090. Date of registration: 08 October, 2010

## Background

Vitamin-D deficiency is considered to be one of the most common nutritional deficiencies and a commonly undiagnosed medical condition in the world. [1] The prevalence of vitamin-D deficiency is 50–90% in the Indian subcontinent. [2] Vitamin-D is primarily produced in the skin after exposure to ultraviolet radiation and less than 10% is derived from dietary sources. [3]

Poor maternal vitamin-D status during pregnancy has been linked to impaired neurodevelopment among adult offspring and to structural changes in the brain such as enlarged lateral ventricles, thinner cortex, and more cell proliferation in animal studies. [4, 5] Human studies have shown that poor vitamin-D status prenatally is associated with adverse neuropsychiatric outcomes including schizophrenia and child autism. [6, 7] Two recent longitudinal studies showed a link between maternal vitamin-D status in early pregnancy and delayed neurocognitive development including language impairment, mental development, and psychomotor development in early childhood. [8, 9] To our knowledge, there are few studies on the association between vitamin-D status in children and neurodevelopment.

Vitamin-D is required for normal calcification of the growth plate and bone mineralization. [10] The major role of vitamin-D in maintaining bone health is to ensure normal calcium and phosphate levels in the blood. Children have higher calcium demands than adults; they require a positive calcium balance to assure adequate calcium for the mineralization of growing bone. [11] It is therefore important to ensure adequate vitamin-D status in order to enhance normal calcification of the growth plate and bone mineralization.

We undertook a randomized controlled trial (RCT) of daily supplementation with folic acid and/or vitamin B12 or placebo for six months in 6 to 30 months old children and measured the 25OHD cocentration in the blood specimens at baseline and at 6 months follow-up. We took the opportunity to estimate the association between baseline and at 6 months follow -up vitamin-D status and neurodevelopment measured by the Ages and Stages Questionnaire-3 (ASQ-3) after 6 months as well as the association between vitamin-D deficiency and physical growth (wasting, stunting and underweight) at baseline and 6 months later.

## Methods

The study was conducted from January 2010 to February 2012 in the low-to-middle socioeconomic neighborhoods of Tigri and Dakshinpuri in New Delhi, India. The total population was around 300,000; details of the population have been described previously [12]. The current analysis has been done within the framework of a randomized double-blind placebo-controlled trial (NCT00717730 at https://clinicaltrials.gov/ct2/show/NCT00717730) involving supplementation with folic acid and/or vitamin B12 or placebo for six months to 1000 children who were 6 to 30 months old at enrollment [12]. Vitamin-D status was available for 960 children at baseline and in 243 at 6 months follow-up. For baseline, our analysis for neurodevelopment is restricted to the 401 children in whom ASQ-3 [13] was administered, for physical growth to 960 children in whom anthropometry data were available at baseline and to 919 children in whom anthropometry data were available 6 months later. Of the 243 children, neurodevelopment data were available for 92 children and anthropometry data were available for all.

### Assessment

Neurodevelopment was assessed 6 months after enrollment using the ASQ-3 which is a developmental screening tool constructed in the USA. [13] The ASQ-3 consists of age-appropriate questionnaires, all containing 30 items divided into five subscales: Communication, Gross motor, Fine motor, Problem-solving and Personal-social, summing up to five subscale scores (range 0 to 60) and a total score (range 0 to 300). The construct and convergent validity of the translated ASQ-3 forms for the current setting were excellent, and our multiple models were able to explain more than 30% of the variability of the ASQ-3 scores. [14]

Details of the Hindi translation and process of validation of ASQ-3, training and standardization methods have been described previously. [15] Three trained field supervisors administered the ASQ-3 directly to the child at the study clinic in the presence of caregivers. The examiners elicited the relevant skills from the child during sessions using standardized materials. The caregiver served as an important contributor in supporting the child, eliciting behaviors and gave relevant information of the child’s development when necessary. During the 11 days of training, the field supervisors were standardized in performing the procedure, and they reached a high inter-observer agreement both during training and in the 10% quality control checks throughout the study. To assess the caregiver’s promotion of child development two questions were selected from the standardized assessment tool Home Observation for Measurement of the Environment (HOME) that were asked the caregivers during the session. [16] One question was on “Mother’s belief that child’s behavior can be modified” and one was on “Mother’s encouragement of developmental advances”.

Trained field supervisors measured weight and length at baseline and after six months of supplementation. Weight was measured to the nearest 50 g using electronic scale (Digitron scale). Length was measured using locally manufactured infantometers reading to the nearest 0.1 cm.

### Analytical procedures

Blood samples were obtained at baseline from all children; 3 mL blood was collected in an evacuated tube containing EDTA (Becton Dickinson). The plasma was centrifuged at ~450×g at room temperature for 10 min, separated, and transferred into storage vials and stored at −20 ^0^ C until analyzed. Plasma concentration of vitamin-D was measured by quantitative electro-chemiluminescence binding assay, with detection of 25 OHD, the hydroxylated forms of vitamin-D2 (Roche Diagnostics, Mannheim, Germany) [17] at the Department of Biochemistry, Christian Medical College, Vellore, India.

### Statistical analysis

Proportions, means (SD) or medians (IQR) were calculated for categorical and continuous variables by vitamin-D status at baseline. Though The Institute of medicine concluded that for maximum bone health a blood level should be at least 20 ng/mL and the Endocrine Society’s Practice Guidelines recommended for maximum bone health a level should be above 30 ng/mL, we considered vitamin-D deficiency was defined at <10 ng/mL (25 nmol/L). [18] We also ran a sensitivity analysis classifying baseline vitamin-D status as <10, 11–20, 21–29 and > = 30 ng/mL.

We used multiple regression and a “purposeful selection of covariates method” to identify variables that were associated with vitamin-D deficiency and our predefined outcomes. [19, 20] These variables were used as adjustment variables in the multiple models where vitamin-D deficiency was the exposure variable. We also examined whether the predefined associations were modified by other variables using interaction terms (on a multiplicative scale) in the multiple regression models.

Multiple linear and logistic regression analyses were used to compare the total ASQ-3 and subscale-scores between the vitamin-D deficient and the vitamin-D non-deficient groups at baseline and at 6 months followup. In logistic regression models the total and subscale ASQ-3 scores were categorized at the 25th percentile. In these models, we adjusted for age of child, mother’s years of schooling, father’s years of schooling, log transformed annual family income, family structure, number of toys in the family, whether or not the family owns books, number of children in the family, hours of play with other children during the week, mother’s belief that child’s behavior can be modified, mother’s encouragement of developmental advances, weight-for-height Z score, weight for-age Z scores and intervention group.

We used multiple linear and logistic regression analyses to measure the association between vitamin-D deficiency and childhood physical growth at baseline and at 6 months follow-up. In logistic regression models, physical growth was categorized as wasting (< −2 Z scores weight-for-height/length), stunting (<−2 z score height/length-for-age) and underweight (< −2 Z scores weight-for-age). In the linear regression models, we used the Z scores of weight-for-height/length (WHZ), height/length-for-age (HAZ), weight-for-age (WAZ) as dependent variables. In these models, we adjusted for age, sex, breastfeeding status, family structure, log transformed annual family income, mother’s years of schooling, father’s year of schooling, baseline level of vitamin B12, folate and anemia status for baseline physical growth as well as, intervention group (placebo, folic acid, vitamin B12, or both) for 6 months later physical growth.

Statistical analyses were performed using STATA version 14 (Stata Corporation, College Station, TX).

We used generalized additive models in the statistical software R version 3.1.2 (The R Foundation for Statistical Computing, Vienna, Austria) to explore nonlinear associations between the vitamin-D status at baseline and HAZ score at baseline after adjustment for potential confounders [21]. We also used generalized additive models to explore nonlinear associations between vitamin-D status at baseline and total ASQ-3 score after 6 months of follow - up.

## Results

A total of 1000 children were included in the main trial. Vitamin-D level was analyzed in baseline samples for 960 (96%) children. Of these, 331 (34.5%) were vitamin-D deficient (<10 ng/ml). The mean (SD) and median (IQR) of vitamin-D level were 14.82 (8.7) ng/ml and 13.15 (8.31, 19.2) ng/ml. The baseline characteristics of the population by vitamin-D status are presented in Table 1. Approximately half of the enrolled children were boys and almost all (98%) were ever breast fed. Approximately 70% of the children were anemic (Hb < 11 g/dl).

**Table 1:** Baseline characteristics of vitamin-D deficient and non-deficient children aged 6–30 months included in the analysis

Characteristics	*n* = 1000	
Number of children for whom samples for vitamin-D were available at baseline	960 (96.0%)	
Proportion of children		
Deficient (< 10 ng/ml)	331 (34.5)	
Non-deficient (≥ 10 ng/ml)	629 (65.5)	
	Deficient	Non-deficient
	*n* = 331	*n* = 629
Infant characteristics		
Age at enrollment, months (mean, SD)	16.9 (7.1)	15.8 (7.0)
Boys	162 (48.9)	328 (52.2)
Ever breastfed	325 (98.2)	622 (98.9)
Anemia (Hb < 11 g/dl)	244 (73.7)	424 (67.4)
Folate, nmol/L (Median, IQR)	9.1 (6.1 to 16.7)	12.5 (6.9 to 21.8)
Vitamin B12, pmol/L (Median, IQR)	266 (181 to 406)	266 (172 to 410)
Socio-demographic characteristics		
Mother’s age, years (Mean, SD)	26.3 (5.8)	25.6 (4.1)
Mother’s schooling, years (Median, IQR)	8 (5,10)	7 (0,10)
Literate mother	257 (77.6)	466 (74.1)
Father’s schooling, years (Median, IQR)	10 (7,12)	9 (6,12)
Annual family income, US dollar(Median, IQR)^a^	1108 (923to 2215)	1292 (923 to2123)

Figures are number (percentages) unless stated otherwise

^a^1 US Dollar = INR 65

The mean (SD) of the total ASQ-3 and subscales scores by vitamin-D status are shown in Table 2. The overall ASQ-3 score was not significantly lower in the vitamin-D deficient group [mean difference − 6.54 (95% CI: -16.15 to 3.08)] compared to the vitamin-D non-deficient group. The Personal social subscale score was significantly lower in the vitamin-D deficient group [mean difference − 2.63 (95% CI: -5.00 to −0.25)], whereas the other subscale scores were not. There was no interaction on a multiplicative scale between intervention (folic acid and/or vitamin B12 or placebo) and vitamin-D deficiency on the total ASQ-3 score or for the subscales scores.

**Table 2:** The association between vitamin-D and total ASQ-3 and subscale scores

	Deficient (Vitamin-D status < 10 ng/ml)	Non-Deficient (Vitamin-D status ≥ 10 ng/ml)	
	Mean (SD) *n* = 165	Mean (SD) *n* = 236	Unadjusted Mean Diff (95% CI)
Total ASQ-3	229.98 (49.76)	236.52 (47.06)	−6.54 (−16.15 to 3.08)
Subscale			
Communication	49.04 (15.19)	49.26 (15.11)	- 0.22 (−3.22 to 2.8)
Gross motor	46.36 (14.39)	48.29 (13.41)	−1.93 (−4.69 to 0.82)
Fine motor	46.73 (14.90)	48.89 (12.09)	−2.15 (−4.81 to 0.50)
Problem-solving	46.42 (13.97)	46.03 (13.45)	0.39 (−2.33 to 3.12)
Personal social	46.42 (12.04)	49.05 (11.84)	−2.63 (−5 to −0.25)*

*p < 0.05

We repeated the analyses using logistic regression after dichotomizing the outcomes at the 25th percentiles. After adjusting for potential confounders, we found a significant difference (OR: 1.63; 95% CI: 1.03 to 2.58) in the Personal-social subscale. The overall ASQ-3 score and the remaining subscales were not associated with vitamin-D deficiency. (Table 3).

**Table 3:** Odds Ratios for the lower quartile of total ASQ-3 and subscale scores compared with Non-deficient vitamin-D status (≥ 10 ng/ml) adjusting for confounders^a^

	Non-Deficient (Vitamin-D status ≥ 10 ng/ml)	Deficient (Vitamin-D status < 10 ng/ml)	
	OR	OR	95% CI
Total ASQ-3	1	1.36	0.79 to2.31
Subscale			
Communication	1	1.58	0.97to 2.59
Gross motor	1	1.27	0.80 to 2.03
Fine motor	1	1.31	0.81to 2.11
Problem-solving	1	1.33	0.84to 2.11
Personal - social	1	1.63	1.03to 2.58*

*p < 0.05

^a^Adjusted for: age of child, mother’s years of schooling, father’s years of schooling, log transformed annual family income, family structure, number of toys in the family, whether or not the family owns books, number of children in the family, hours of play with other children during the week, mother’s belief that child’s behavior can be modified, mother’s encouragement of developmental advances, weight-for-height Z score, weight for-age Z scores, anemia status at baseline and intervention group

Tables 4 and 5 show the distribution of the proportion of children stunted, wasted and underweight according to vitamin-D status at baseline and 6 months later respectively. At baseline in the vitamin-D deficient group, the proportion of stunted, wasted and underweight children was 35.4%, 10.6% and 30.5% respectively. 6 months later, in the vitamin-D deficient group the proportion of stunted, wasted and underweight children was 37.8%, 13.9% and 32.8% respectively. Vitamin-D deficiency was not associated with stunting nor wasting nor underweight at baseline or 6 months later in this population.

**Table 4:** The association between baseline Vitamin-D status and growth (at baseline) among children

	Deficient (Vitamin-D status < 10 ng/ml) n = 331	Non-Deficient (Vitamin-D status ≥ 10 ng/ml) n = 629	Adjusted β coefficient (95% CI)^a^
Z scores: Mean(SD)			
HAZ	−1.56 (1.24)	−1.63 (1.16)	.05 (−0.10 to 0.20)
WHZ	−0.86 (0.92)	−0.89 (0.94)	.02 (−0.10 to 0.14)
WAZ	−1.46 (1.06)	−1.52 (1.05)	.03 (−0.10 to 0.17)
			Adjusted OR (95% CI)^a^
Stunted, n (%)	117 (35.4)	233 (37.0)	0.84 (0.63 to 1.13)
Wasted, n (%)	35 (10.6)	68 (10.8)	0.99 (0.64 to 1.54)
Underweight, n (%)	101 (30.5)	197 (31.3)	0.91 (0.67 to 1.23)

^a^Adjusted for: age, sex, breastfeeding status, log transformed annual family income, family structure, mother’s years of schooling, father’s years of schooling, baseline levels of vitamin B12, folate, anemia status at baseline

**Table 5:** The association between baseline Vitamin-D status and growth (6 months later) among children

	Deficient (Vitamin-D status < 10 ng/ml) *n* = 323	Non-Deficient (Vitamin-D status ≥ 10 ng/ml) *n* = 596	Adjusted β coefficient (95% CI)^a^
Z scores: Mean(SD)			
HAZ	−1.69 (1.17)	−1.81 (1.12)	0.11 (−0.20 to 0.43)
WHZ	−0.93 (0.97)	−0.95 (0.92)	0.20 (−0.05 to 0.46)
WAZ	−1.55 (1.08)	−1.62 (1.00)	0.20 (−0.06 to 0.47)
			Adjusted OR (95% CI)^a^
Stunted, n (%)	122 (37.8)	265 (44.5)	0.81 (0.61 to 1.07)
Wasted, n (%)	45 (13.9)	67 (11.2)	1.35 (0.90 to 2.04)
Underweight, n (%)	106 (32.8)	209 (35.1)	0.97 (0.72 to 1.29)

^a^Adjusted for: age, sex, breastfeeding status, log transformed annual family income, family structure, mother’s years of schooling, father’s years of schooling, baseline level of vitamin B12, folate, anemia status at baseline, intervention group

No substantial or significant differences were found when we did these analyses using HAZ, WHZ and WAZ scores as outcome variables both at baseline and 6 months later i.e. vitamin-D deficiency is not associated with any of the physical growth parameters on a continuous scale.

We estimated vitamin D status at end study in only 243 children, and the prevalence of deficiency was 38.3%. Of the 243 children neurodevelopment data were available for 92 children. None of the neurodevelopment outcomes (Total ASQ-3, sub scales of ASQ-3 i.e. Communication, Gross motor, Fine motor, Problem - solving, Personal- social), nor any of the grwoth outcomes were significantly associated with vitamin D status after 6 months (Not shown in table).

We also did a sensitivity analysis by classifying baseline vitamin-D status as <10, 11–20, 21–29 and > = 30 ng/mL. 34.6% children were <10, 42.4% were 11–20, 17% were 21–29 and 6% were above 30 ng/ml. None of the neurodevelopment outcomes (Total ASQ-3, subscales of ASQ-3 i.e. Communication, Gross motor, Fine motor, Problem solving, Personal- social) and growth outcomes (wasting, stunting and underweight at baseline and end study) were significantly associated with any of these quartiles.

**Fig. 1:**
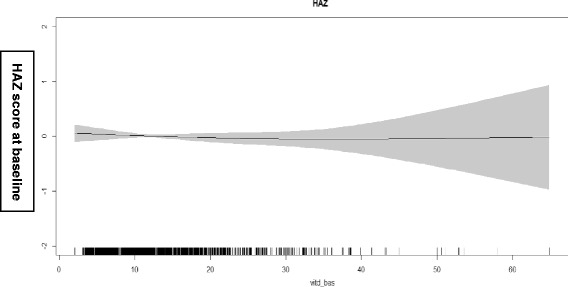
Associations between vitamin-D level at baseline and HAZ score at baseline. The graphs were constructed using generalized additive models in R, the solid line depicts the association of vitamin-D level at baseline and HAZ score at baseline. The shaded area spans the 95% confidence interval of this association

**Fig. 2:**
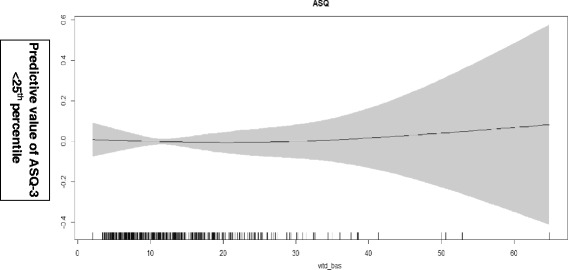
Associations between vitamin-D level at baseline and total ASQ-3 score after 6 months of follow up. The graphs were constructed using generalized additive models in R, the solid line depicts the association of vitamin-D level at baseline and total ASQ-3 score. The shaded area spans the 95% confidence interval of this association

The association between vitamin-D status at baseline and HAZ score at baseline and total ASQ-3 score after 6 months are depicted in Figs 1 and 2. There was no association.

## Discussion

Except for the Personal - social scale, we did not find any significant differences in the total ASQ-3 and subscale scores between vitamin-D deficient and non-deficient children. Nor did we find any association between growth indices and vitamin D deficiency.

Our findings are consistent with those from a recently conductedcohort study in India, where vitamin-D status was not associated with the gross motor subscale of ASQ-3 among school- aged children [22]. A recent prospective study also suggests an inverted-U–shaped relation between neonatal vitamin-D status and neurocognitive development in toddlers [23]. Recent studies have shown an association between prenatal vitamin-D deficiency with delayed language development in early childhood [24, 25]. However, the association between vitamin-D deficiency and the Personal social subscale may very well be a chance finding considering the number of outcomes examined. It should also be noted that the personal social scores is the domain with poorest psychometric properties. [15]

The ASQ-3 is a screening tool for the assessment of developmental delay constructed in the US with binary cut-offs. It has however been used to measure developmental status on a continuous scale in the present study as in several other studies. [13] Alpha values indicated questionable internal consistency in a few subscales and age categories. Poor internal consistency can be due to constant items (lack of variability of the responses) or random error which may result in false negative results (type II error). However, in another publication from this study we also found that the ASQ-3 scores were associated with linear and ponderal growth, the incidence of diarrhea and pneumonia, as well as socioeconomic status and stimulation and learning opportunities. In fact, these variables explained more than 30% of the variability of the ASQ scores which demonstrates that the translated ASQ-3 test had good convergent validity. [14] Thus, the lack of findings of an association between vitamin-D and ASQ-3 scores is probably not due to poor psychometric properties of the test. In the translated ASQ-3 version, the standardized alphas for the total ASQ-3 scores were strong, indicating an overall acceptable internal consistency.

Type II errors can also be due to low sample size, negative confounding, and/or weaknesses with the immunological vitamin –D assay. We used an immunological method to measure vitamin-D concentration. It should be noted that immunoassays can overestimate 25OHD [26] because it is lipophilic which makes it vulnerable to matrix effects in the protein binding assays. [27]

We found no association between vitamin-D deficiency and physical growth at baseline and 6 months later. Similar findings have been described in pre-school children in Nepal and HIV exposed but uninfected infants in Africa, where vitamin-D deficiency was not associated with stunting and underweight, although wasted children have been found to be more commonly vitamin- D deficient. [28, 29] However, a recent trial in India showed that weekly administration of 1 RDA of vitamin-D supplementation for six months among LBW infants significantly increased weight and length, and decreased the proportion of children with stunted growth. [30] In addition, an observational study of Canadian infants found higher vitamin - D concentrations during infancy to 3 years of age were associated with leaner body composition. [31]

Although the major role of vitamin-D in maintaining bone health is to ensure normal calcium and phosphate levels in the blood, [32] we did not find any association between vitamin-D deficiency and ponderal or linear growth.

There may be deficiencies other growth-limiting macro and micronutrients such as calcium, zinc, vitamin –B12 etc. Poor quality of food i.e. lower proportion of animal source protein may also contribute to poor growth. The influence of vitamin D might be small in the light of other growth limiting factors.

To our knowledge this is the first analysis to examine the association between vitamin-D status and several domains of the ASQ-3. The strengths of our study are that the data are from a well conducted study with very low attrition rates. [33] More comprehensive assessment tools, such as the Bayley scales, or tools for social emotional functioning, could have added a broader picture of the children’s skills and abilities yielding different results. There was a need for follow up assessments since 6 months may not be sufficient to assess the associations between vitamin- D status and development and growth. Finally, more advanced neuroimaging techniques may have identified unique changes to the developing brain in early childhood that could have been linked to vitamin-D deficiency.

## Conclusion

The results from this analysis do not support that vitamin-D deficiency in early childhood is important for growth and neurodevelopment.

## Abbreviations

25OHD: 25-hydroxy-vitamin-D
ASQ-3: Ages and Stages Questionnaire
CI: Confidence Interval
HAZ: Height/length-for-age Z score
Hb: Hemoglobin
HOME: Home Observation for Measurement of the Environment
IQR: Inter quartile range
OR: Odds ratio
RCT: Randomized controlled trial
RDA: Recommended Daily Allowance
SD: Standard Deviation
WAZ: Weight-for-age Z score
WHZ: Weight-for-height/length Z score
